# Compact adaptive spectral imager enabled by MEMS Fabry-Perot filtering chip in longwave infrared

**DOI:** 10.1038/s41378-026-01300-6

**Published:** 2026-05-26

**Authors:** Kui Zhou, Xiaodong Wang, Geng Tong, Xingchen Xiao, Jiancun Zhao, Xiaochang Yu, Yiting Yu

**Affiliations:** 1 School of Mechanical Engineering, Ningbo Institute of Northwestern Polytechnical University, Key Laboratory of Micro/Nano Systems for Aerospace (Ministry of Education), Key Laboratory of Micro- and Nano-Electro-Mechanical Systems of Shaanxi Province, Northwestern Polytechnical University, Xi’an, China; 2 Key Laboratory of Scale Manufacturing Technologies for High-Performance MEMS Chips of Zhejiang Province, Key Laboratory of Optical Microsystems and Application Technologies of Ningbo City, Ningbo, China

**Keywords:** Applied optics, Other nanotechnology

## Abstract

Longwave infrared (LWIR) spectral imaging offers unique capabilities for gas/liquid detection, mineral exploration, environmental monitoring, and military security applications. Conventional LWIR spectral imagers, however, are hampered by reliance on existing dispersion elements, resulting in inflexible operation, limited spectral channels, and excessive bulk, which preclude deployment in intelligent, multi-scenario settings. To address such an issue, we introduce a compact adaptive spectral imager (CASI) leveraging a LWIR large-aperture micro-electro-mechanical systems based Fabry-Perot filtering chip (MEMS-FPFC). A modular chip-front optical architecture yields an ultra-compact volume (150 × 77 × 88 mm³), low-weight (1.11 kg), and “plug-and-play” operation. A synergistic adaptation control system exploits the intrinsic programmability of the MEMS-FPFC to enable diverse adaptive imaging modes-including programmable spectral acquisition spanning global coarse/fine scans, localized fine scans, and arbitrary spectral band combinations-tailored to specific application demands. This adaptability, coupled with spectral channel selection and image fusion algorithms, facilitates efficient and precise target identification. The CASI’s compactness and programmability enable integration onto small unmanned platforms, advancing intelligent LWIR spectral imaging for field-deployable applications in industrial pollution monitoring, mineral prospecting, and security.

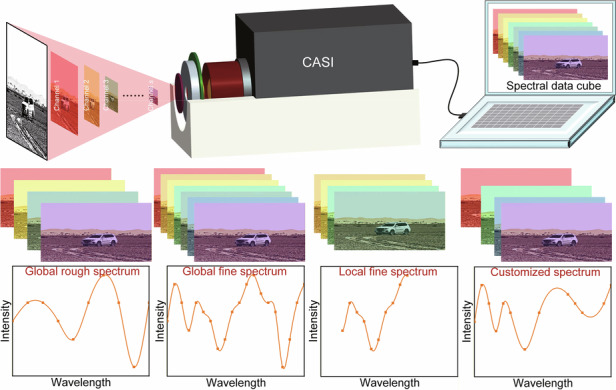

Longwave infrared (LWIR) spectral imaging combines the three-dimensional (3D) target characterization of integrated spatial and spectral information, with the inherent all-weather, all-time advantages of thermal infrared sensing^1^. This dual capability enables widespread adoption in gas/fluid detection^2,3^, mineral identification^4^, environmental monitoring^5^, and military reconnaissance^6,7^. By fusing multidimensional datasets, the technique facilitates intelligent extraction of physicochemical properties from imaged targets, advancing applications in automated monitoring and intelligent perception. The dispersion element fundamentally determines the performance envelope of spectral imager, dictating spatial-spectral sampling characteristics and engineering constraints^8,9^. State-of-the-art LWIR spectral imagers primarily implement following spectral sampling methods: (i) wavelength selective filters^10,11^, (ii) spatial dispersion optics^12–15^, or (iii) interferometric modulation^16–18^. Although commercially available LWIR spectral imager exist, their architectures necessarily compromise between competing performance metrics^19,20^. For quantitative analysis demanding high spectral resolution, diffraction-grating and Fourier-transform LWIR spectral imager currently represent the benchmark solutions, delivering hyperspectral imaging capabilities^21^. Yet their dependence on precision mechanical scanning mechanisms introduces fundamental limitations-bulky system architectures and inherently low temporal resolution from sequential wavelength acquisition. For qualitative analysis and spectral target detection algorithms, optimal performance can be achieved through strategic fusion of selected spectral channels^22,23^. Conventional hyperspectral imagers introduce significant data redundancy in these applications, whereas multispectral imagers based on customized wavelength selective filters^24^ and emerging micro-electro-mechanical systems (MEMS) multiband filter^25–27^ enable efficient acquisition of diagnostically relevant spectral features. The integration of computational spectroscopy with multispectral imaging enables simultaneous high-efficiency data acquisition and high-fidelity spectral reconstruction, representing a paradigm shift in spectral sensing technology^28–30^. The application-specific nature of these optimized spectral channels creates an implementation dilemma: while spectral channel expansion could improve detection versatility, it comes at the prohibitive cost of further increasing the mechanical dimensions of already bulky filter wheel systems or MEMS process complexity. The widespread adoption of LWIR spectral imaging has been fundamentally constrained by inherent system limitations.

Overcoming these barriers demands a transformative approach through the development of miniaturized, dynamically tunable optical filters-a technological breakthrough that would enable compact adaptive LWIR spectral imaging platforms capable of addressing diverse application scenarios. Combining MEMS based Fabry-Perot filter chips (MEMS-FPFC) with advances in computational optics enables novel spectral imager with intelligent capabilities^31^. These chips employ multi-beam interference principles and MEMS actuation strategy to achieve selective tunable spectral filtering^32^. Programmable electrical actuating parameters allow adaptive spectral filtering, while the synergy between tunable hardware and software algorithms leverages flexible spectral control. This integrated approach equips spectral imager with programmable and adaptive functionalities, enabling their transition from “fixed-configurations” to “intelligent-adaptation”. Capitalizing on the advantages of MEMS-FPFC, research institutions globally have pursued its application in spectral imaging^33^. Notable leaders in this field include VTT Technical Research Centre of Finland^34–39^ and Unispectral Ltd^40,41^. These pioneers have developed MEMS-FPFCs employing diverse actuation methods, enabling compact spectral imagers for unmanned aerial vehicle (UAV), nanosatellites, and commercial use, demonstrating core spectral imaging functionality. However, current spectral imagers fall short of fully exploiting MEMS-FPFC’s potential. Limitations inherent in filtering methodologies and control schemes hinder their ability to achieve adaptive spectral imaging. Additionally, existing research primarily focuses on the visible to near-infrared spectrum, with a notable absence of studies on LWIR MEMS-FPFC and their spectral imager. Consequently, harnessing the inherent advantage of MEMS-FPFC, its flexible tunability, through the development of programmable, adaptive control systems, represents the pivotal challenge for realizing adaptive spectral imaging functionality.

In our previous work, we addressed the initial challenge by developing an 11-mm aperture electromagnetically actuated LWIR MEMS-FPFC^42^. This device achieves bidirectionally and linearly modulation filtering across the 8-12 μm wide spectral range, with subsequently implementation of programmable filtering functionality. This capability underpins our novel compact adaptive spectral imager (CASI) in LWIR, whose system architecture, adaptive functionality, and validation are detailed herein. We establish CASI’s principial structure and functional requirements for diverse scenarios, elaborating on the MEMS-FPFC’s critical programmable filtering performance, and develop a synergistic adaptation control system to realize adaptive imaging. Following spectral and radiometric calibration, we validate the CASI’s adaptive functionality in targeted applications. The CASI platform achieves multi-scenario operation (“one instrument, multiple uses”), supports deployment on lightweight platforms like small UAVs, and holds significant promise for biomedical diagnostics, gas/liquid sensing, and mineral exploration.

## Results and discussions

### System integration of CASI

#### Working principle of CASI

**Fig. 1:**
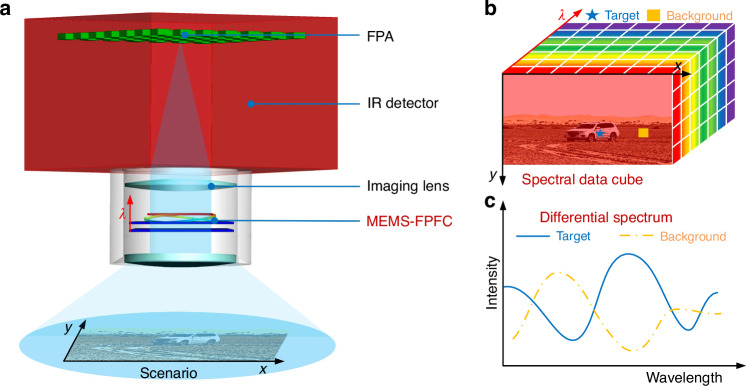
The schematically system configuration and working principle of spectral imager based on MEMS-FPFC. **a** the system configuration of CASI, which consists of MEMS-FPFC, imaging lens and IR detector. **b** The spectral data cube of spectral images obtained by CASI. **c** The differential spectral signatures of scenario extracted from the spectral data cube

The fundamental architecture of CASI is illustrated in Fig. 1a, comprising the MEMS-FPFC, an imaging lens and an infrared (IR) detector. Serving as the core spectral dispersion element, the MEMS-FPFC disperses scenario radiation transmitted through the imaging lens into a series of narrowband spectral channels. These spectral channels are sequentially imaged by the IR detector, ultimately forming a spectral data cube containing the spectral information of the target scenario (Fig. 1b). Processing this data cube enables extraction of characteristic spectral signatures from both targets and backgrounds (Fig. 1c). The volume of spectral image data within the cube-along with spectral parameters such as resolution and waveband ranges-is determined by the spectral channels partitioned by the MEMS-FPFC. For applications requiring detailed spectral information (high spectral resolution), the MEMS-FPFC performs fine spectral scanning. The resulting high-resolution data facilitates quantitative analysis of material properties and supports training of spectral channel fusion algorithms. Conversely, when multispectral imaging suffices (lower spectral resolution), the MEMS-FPFC executes coarse spectral scanning or programmed filtering according to predefined spectral channels. This configuration enables qualitative material analysis or targeted fusion detection within specific spectral channels. Critically, customization of the MEMS-FPFC’s filtering modes achieves “one system, multiple modalities” functionality, thereby overcoming the fixed operational limitations inherent in conventional spectral imagers.

Achieving programmable adaptive spectral imaging in the LWIR regime requires addressing two critical challenges: (ⅰ) developing a large-aperture LWIR MEMS-FPFC with superior filtering performance, and (ⅱ) establishing an adaptive control system capable of programming spectral image acquisition through coordinated operation of the MEMS-FPFC and the IR detector. While the former has been resolved in our prior research, this work focuses on implementing the latter to realize fully programmable adaptive spectral imaging.

#### Programable LWIR MEMS-FPFC

**Fig. 2:**
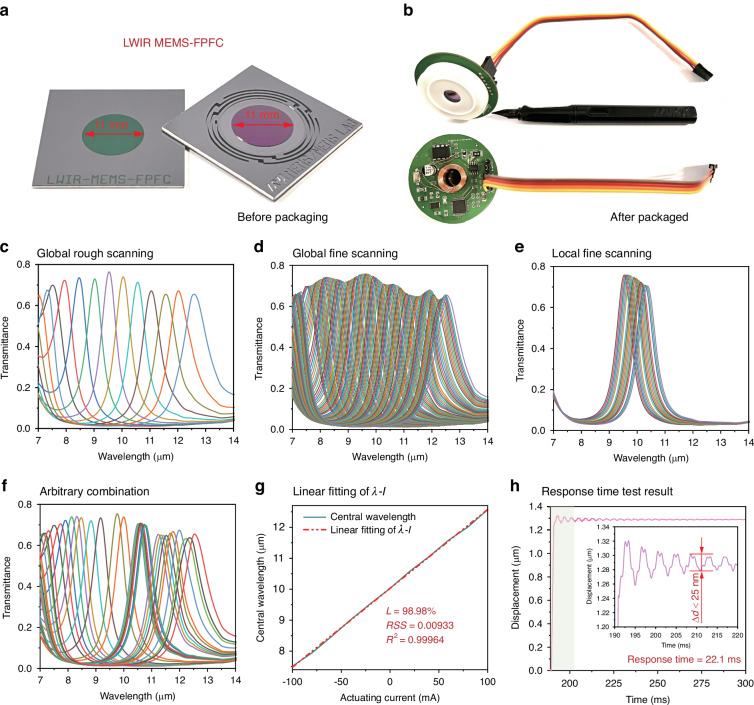
The LWIR MEMS-FPFC and its programable filtering tunability. **a** the 11-mm large aperture LWIR electromagnetically actuated MEMS-FPFC before packaging. **b** After packaged on PCB, the LWIR MEMS-FPFC becomes an independent filtering module; **c**–**f** By programming the actuating current PWM signal, the MEMS-FPFC behaves different filtering mode including global rough scanning, global fine scanning, local fine scanning and arbitrary spectral channel combination filtering mode; **g** After fitting, the *λ-I* show a linear relationship; **h** the response time test result

The MEMS-FPFC serves as the core dispersion component for achieving adaptive spectral imaging. As presented in Fig. 2a, the electromagnetically actuated MEMS-FPFC developed in our previous work features the largest aperture size in LWIR waveband of 11 mm, ensuring high energy and information throughput for spectral imaging. After packaging, the MEMS-FPFC becomes an independent filtering module (Fig. 2b), facilitating efficient modular integration of the CASI with a “plug-and-play” capability. Upon further research, the packaged MEMS-FPFC employs pulse width modulation (PWM) for actuating and filtering, enabling programmable current control through PWM signal programming, thereby achieving programmable filtering. The filtering performance of the MEMS-FPFC under different modes was tested using a Fourier-transform infrared spectrometer (FTIR), with results shown in Fig. 2c–f. By programming the actuating current, the MEMS-FPFC can operate in multiple filtering modes, including coarse scanning (Fig. 2c: 20 mA current step), fine scanning (Fig. 2d, e: 2 mA current step), and arbitrary spectral channel combinations (Fig. 2f), meeting the application requirements of CASI for multispectral, hyperspectral, and customizable spectral channel imaging. Additionally, within an actuating current range of -100–100 mA, the MEMS-FPFC achieves broadband tunable filtering across 7.502–12.542 μm. The linear relationship between the filtering wavelength (*λ*) and actuating current (*I*) was fitted as:

1 $$\lambda =0.0225\ast I+10.029$$

As shown in Fig. 2g. Although the MEMS-FPFC is subject to unavoidable filtering errors (Δλ) arising from environmental factors and actuating current fluctuations, we introduce linearity (*L*) as a metric to evaluate such errors. Based on the specified wavelength error (Δ*λ*) of ±80 nm for commercial LWIR narrow-bandpass filters^43^, the MEMS-FPFC is designed with a linearity exceeding 98%, corresponding to Δ*λ* < 80 nm across the 8–12 μm waveband. In actual, the fitted *L* was 98.98%, with residual sum of squares (*RSS*) and coefficient of determination (*R²*) values of 0.009 and 0.999, respectively, indicating excellent linear filtering performance. This linear relationship ensures precise and controllable programmable filtering, with a maximum acceptable filtering error of 51.41 nm within the 7.502–12.542 μm operating spectral range. Furthermore, the response time of the MEMS-FPFC was evaluated under maximum actuating current (100 mA). As shown in Fig. 2h, the displacement exhibits a damped oscillatory response before settling. We defined the response time as the duration required for the amplitude to decay below 25 nm, which corresponds to a filtering error of 50 nm. Based on this criterion, the response time was determined to be 22.1 ms.

To ensure stable filtering performance across various spectral imaging modes and application scenarios, we conducted a comprehensive evaluation of the filtering stability of the MEMS-FPFC. The tests mainly included repeatability measurements and an assessment of environmental influences. Detailed procedures and results are provided in Supplementary Information 1. The repeatability tests revealed a maximum wavelength error of 54 nm. Although this value slightly exceeds the single-measurement result of 51.41 nm, it remains within the design linearity tolerance of the MEMS-FPFC. The maximum transmittance error was 0.6%. Given the transmittance range of the MEMS-FPFC (63.8–76.2%), this absolute error corresponds to a relative error of less than 1%. Under this condition, the resulting radiometric calibration uncertainty remains below 1% and can therefore be considered acceptable.

Regarding environmental factors, thermal drift tests showed that within the linear thermal-expansion range, the wavelength shift (Δ*λ*) varies linearly with temperature (*T*), contributing a maximum filtering error of 39.9 nm. Forced-vibration tests identified a first resonant frequency of 212.4 Hz. Under 0–180 Hz vibrations, the maximum displacement amplitude remained below 24.5 nm, leading to a wavelength error of less than 49 nm. All errors induced by these environmental factors lie within the linearity error margin of the MEMS-FPFC. In addition, based on the FP operating principle, the incident angle of light also shifts the filtered wavelength. Tests over incidence angles of 0–15° confirmed a blue shift with increasing angle. At 14° incidence, the blue shift measured across the 8–12 μm band reached 236.31–358.81 nm. These systematic stability tests provide a solid experimental foundation for the construction and practical deployment of the CASI system. Furthermore, to mitigate the aforementioned errors, we are developing a closed-loop feedback control method for the MEMS-FPFC. By incorporating real-time compensation, this approach enables more precise spectral tuning, which is expected to further reduce wavelength inaccuracies beyond the current ±80 nm tolerance.

#### Optical system integration

**Fig. 3:**
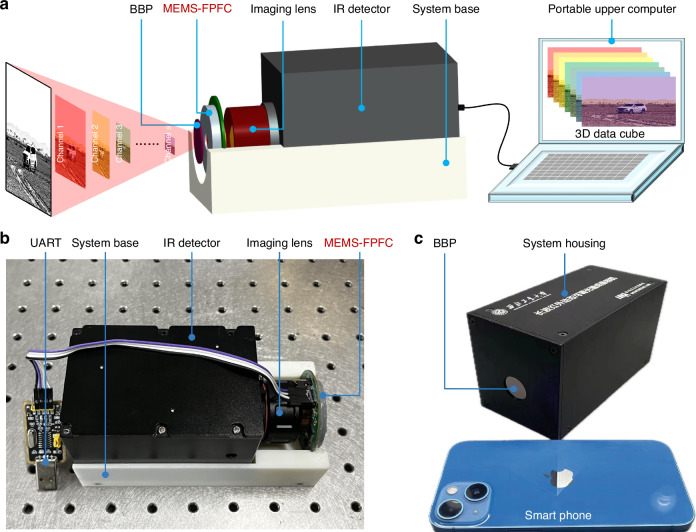
The system configurations of CASI. **a** the modular system configurations of CASI. **b** The interior configurations. **c** the fully integrated CASI has a compact volume as smart phone

Based on the fundamental structural principles of CASI outlined in Fig. 1a and the filtering characteristics of the LWIR MEMS-FPFC, we propose the basic architecture of CASI shown in Fig. 3a. This system employs a chip-front configuration where the MEMS-FPFC is positioned ahead of the optical path rather than between the imaging lens and IR detector. This configuration offers three primary advantages: it mitigates wavelength shift caused by large incident angles, enables modular assembly for “plug-and-play” MEMS-FPFC operation, and shortens the optical path to take the benefits of the large-aperture MEMS-FPFC technology, thereby enhancing both energy throughput and spectral data acquisition capacity. Beyond the core components of imaging lens, IR detector, and MEMS-FPFC, the system incorporates a broad bandpass filter (BBP, OD5, 8.0–12.8 μm bandpass) that serves dual purposes: eliminating out-of-band stray light to ensure monochromatic light acquisition while simultaneously acting as a protective optical window for internal components.

To achieve system miniaturization, a customized uncooled vanadium oxide (VOₓ)-based IR detector with a resolution of 640 × 512 pixels was selected, integrated with a 13 mm-focal-length imaging lens. The detector dimensions match the aperture size of the MEMS-FPFC, with its key parameters detailed in Table 1. Electronic control is implemented through a compact portable upper computer that synchronized adaptive programming of both the MEMS-FPFC and focal plane array (FPA), enabling the system’s programmable adaptive spectral imaging functionality. With the abovementioned selections, the system integration process is shown in Fig. 3b, c according to the designed modular system configurations. The IR detector and MEMS-FPFC are assembled with the system base by transition fit, ensuring the robust system structure and “plug and play” function. After the protective light-weight aluminum alloy system housing assembled with the system base, the fully integrated CASI boasts a small volume of 150*77*88 mm^3^ and a light wight of 1.11 kg, enabling future loading on the lightweight and compact UAVs.

**Table 1:** The key specifications of selected uncooled IR detector

Specifications	Index
Type of detector	Uncooled vanadium oxide (VOₓ)-based detector
Response range	8–12 μm
Resolution	640*512
Imaging lens	13-mm focal length
Maximum FPS	25 Hz
Dynamic range	8–16 bit
Noise equivalent temperature difference	50 mK
Digital output port	GigE
Power	12 V/DC

#### Strategy of adaptive spectral imaging

**Fig. 4:**
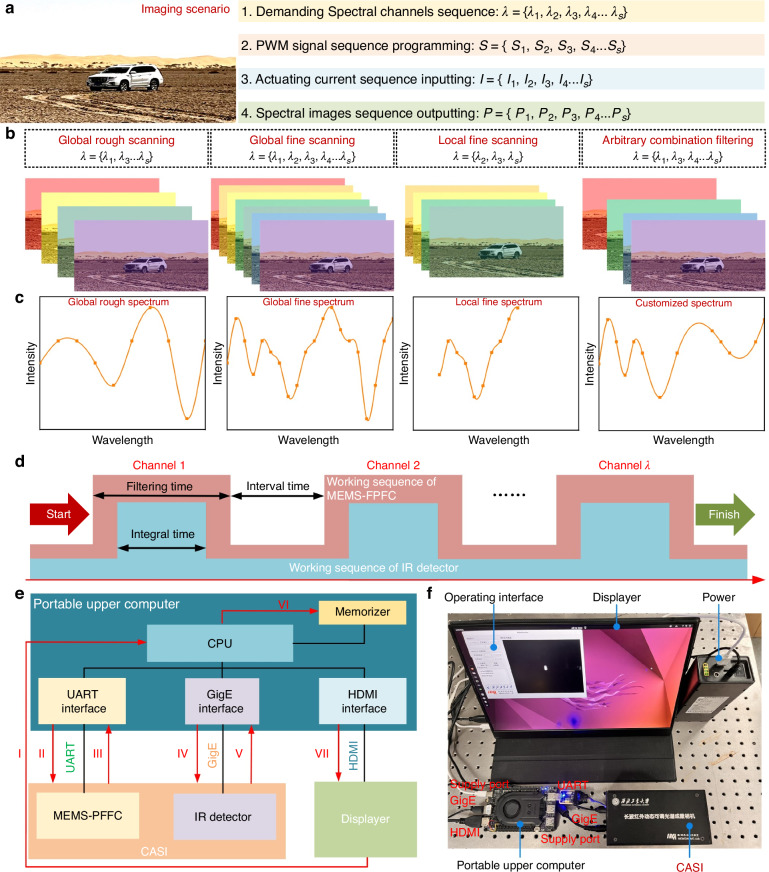
The programmable spectral imaging functional schematics and calibrations of CASI with synergistic control system. **a** The adaptive spectral imaging workflow based on the programmable filtering functionality of MEMS-FPFC. **b**, **c** The spectral data cubes and signature spectrums under different imaging modes. **d** Synchronization mechanism of the CASI. **e** The schematic of the programmable, adaptive spectral imaging process based on collaborative matching. **f** The CASI prototype with integrated electronic systems

By utilizing the MEMS-FPFC’s multi-mode programmable filtering advantages, the CASI can transcend the limitations of conventional spectral imagers to attain adaptive spectral imaging, as illustrated in Fig. 4a. Taking the global fine scanning as an example, high-resolution spectral information of a new scenario is acquired firstly for quantitative analysis or training spectral imaging detection algorithm. This requires programming sequences (***λ***) for all spectral channels:

$${\boldsymbol{\lambda }}=\{{\lambda }_{1},{\lambda }_{2},{\lambda }_{3},{\lambda }_{4}\ldots {\lambda }_{s}\}$$

Based on the fitted *λ-I* relationship, the required actuating current sequences (***I***) are calculated:

$${\boldsymbol{I}}=\{{I}_{1},{I}_{2},{I}_{3},{I}_{4}\ldots {I}_{s}\}$$

Subsequently, the corresponding PWM actuating signal sequences (***S***) are programmed in the host computer:

$${\boldsymbol{S}}=\{{S}_{1},{S}_{2},{S}_{3},{S}_{4}\ldots {S}_{s}\}$$

The MEMS-FPFC then performs scan filtering according to the current sequence ***I*** while the IR detector captures sequential images to generate the spectral image sequences (***P***):

$${\boldsymbol{P}}=\{{P}_{1},{P}_{2},{P}_{3},{P}_{4}\ldots {P}_{s}\}$$

as shown in Fig. 4b, c. Analysis of this sequence yields detailed spectral information for quantitative analysis and training of spectral imaging detection algorithms. However, global fine scanning requires substantial time, limiting efficiency for rapid spectral imaging. For scenarios demanding quick qualitative analysis, the same spectral channel programming approach enables rapid global coarse scanning or localized fine scanning. Notably, once characteristic spectral channels of a target are identified, spectral imaging can be selectively programmed for arbitrary combinations of these spectral channels. This captures only effective characteristic spectral channel image sequences, minimizing data redundancy while enabling efficient target detection through spectral image fusion algorithms. Empowered by the MEMS-FPFC’s programmable filtering, the CASI dynamically responds to a range of spectral imaging requirements, facilitating efficient adaptive spectral imaging.

Since both the MEMS-FPFC and the infrared detector are spatiotemporally modulated devices, their timing must be synchronized to acquire valid spectral image data. The synchronization mechanism is illustrated in Fig. 4e. Taking a single spectral channel as an example, the MEMS-FPFC requires a settling time (*τ*₁) to reach a steady state, followed by a holding period (*τ*₂) during which filtering occurs. Within this interval, the infrared detector integrates signal over a predefined exposure time (*τ*₃) to capture the spectral image for that channel. Once acquisition for a given channel is complete, the MEMS-FPFC transitions to the next channel over a settling interval, and the acquisition sequence is repeated. By executing this timing sequence channel by channel, the complete spectral data cube is obtained. Moreover, the imaging frame rate of the CASI system can be customized through programmable control of each timing parameter. Based on the measured response time of the MEMS-FPFC (22.1 ms, Fig. 2g), *τ*₁ must exceed this value. Given that the maximum frame rate of the infrared detector is 25 Hz, *τ*₂ is required to be greater than 40 ms. In practice, we set *τ*₁ = 40 ms and *τ*₂ = 60 ms, resulting in an imaging frame rate of 10 Hz for the CASI. At this frame rate, the CASI is primarily intended for static or quasi-static scenarios.

To enable above adaptive spectral imaging, we developed a programmable synergistic adaptation control system for CASI, as illustrated in Fig. 4e, achieving precise temporal synchronization between the MEMS-FPFC and the IR detector for accurate spectral image acquisition. The field-deployable hardware integrates a Linux-embedded upper computer, CASI, and portable displayer. The upper computer orchestrates the MEMS-FPFC and IR detector synchronization via UART and GigE interfaces, while an HDMI-connected displayer enables operational monitoring. The host computer implements programmed control logic to coordinate the MEMS-FPFC and the IR detector through dual communication protocols: UART for filter tuning commands and GigE for high-speed image transfer. As detailed in Fig. 4d, the basic operational workflow initiates with (I) spectral acquisition algorithms generating pulse-width modulation (PWM) signals. (Ⅱ) These signals generate actuating current sequences in the MEMS-FPFC’s electromagnetic coil, actuating filter elements to target spectral channels (Ⅲ) while feedback signals verify positioning. (Ⅳ) Synchronized detector capture is subsequently triggered, (Ⅴ) with acquired spectral images transferred to the host for (Ⅵ) storage and (Ⅶ) display visualization. Iterative execution across programmed spectral channels constructs full spectral data cubes. Component selection (Fig. 4f) prioritizes light-weight UAVs deployment compatibility, featuring a compact Linux host board with versatile peripherals. The integrated system operates at <10 W power consumption, supported by a 12 V external power supply.

### System calibration

Accurate spectral signature extraction capability of the CASI necessitates both spectral and radiometric calibration. Detailed calibration procedures, data processing steps, and the corresponding results are provided in Supplementary Information 2. Spectral calibration was achieved using a monochromator, by comparing discrete spectral channels within 8–12 μm waveband against the monochromator’s precisely defined output wavelengths, efficient and accurate calibration was attained. Radiometric calibration, involving noise correction, non-uniformity correction, and absolute radiometric calibration, enables the extraction of spectral feature information from the spectral data cube. The post-calibration validation using standard blackbody radiation targets confirms precise spectral feature extraction, as evidenced by the close agreement between measured data and theoretical Planck curves.

Upon completion of the calibration protocol, comparative analysis with commercial LWIR spectral imagers (Table 2) demonstrates CASI’s advantages: its modular design achieves a 28.07° × 22.62° field-of-view (FOV) and an instantaneous FOV spatial resolution of 0.77 mrad. In Section 2.1, which analyses the angular dependence of the MEMS-FPFC, incident angles up to 14° are shown to induce considerable wavelength shifts, thereby compromising spectral accuracy. Accordingly, during operation of the CASI prototype, we preferentially select distant targets so that light enters the system in a near-collimated form and is imaged near the centre of the field of view. In future work, the optical design of the CASI will be further optimized to reduce sensitivity to incident-angle variations. In addition, via the broadband linear filtering capability, the MEMS-FPFC empowers the CASI to achieve up to 161 programmable spectral channels (surpassing alternatives in the 8–12 μm range), which enables flexible spectral resolution customization and adaptive imaging modalities. The integration of uncooled IR detector and miniaturized MEMS filter yields a compact system, meeting deployment requirements for light-weight UAV platforms and facilitates the practical field deployment of long-wave infrared spectral imaging in the future. However, Commercial long-wave infrared spectral imaging systems typically employ cooled detectors with higher responsivity and are coupled with high-throughput optical designs. Although these systems are larger and heavier, they offer superior imaging performance. Even though we have not found reported values for their noise-equivalent temperature difference (NETD) in the available literature, the CASI prototype achieves an NETD of 1.066–1.542 °C in the 8–12 μm waveband. Future efforts will be directed toward optimizing the CASI system design, with the goal of enhancing imaging performance for practical deployment.

**Table 2:** Performance comparison between CASI and commercial LWIR spectral imagers

Performance	CASI	Telops hyper-CAM mini	Telops MS V1kx
Dispersion element	LWIR MEMS-FPFC	Michelson interferometer	8-channels filtering wheel
Spatial resolution	640*512 (IFOV = 0.77 mrad)	320*256	640*512
Waveband (μm)	8-12	7.4–11.8	7.5–11.5
Spectral channels	161	-	8
Spectral resolution (nm)	Customizable (minimum sampling stepwise 22.5 nm)	66.6–400	-
NETD (°C)	1.066–1.542	-	-
FOV	28.07° × 22.62°	13.5° × 10.9°	-
Weight (kg)	1.11	<20	<13
Volume (cm^3^)	15 × 7.7 × 8.8	28 × 35 × 38	35.2 × 21.6 × 23.6

### Spectral imaging verification

#### Adaptive spectral imaging verification

**Fig. 5:**
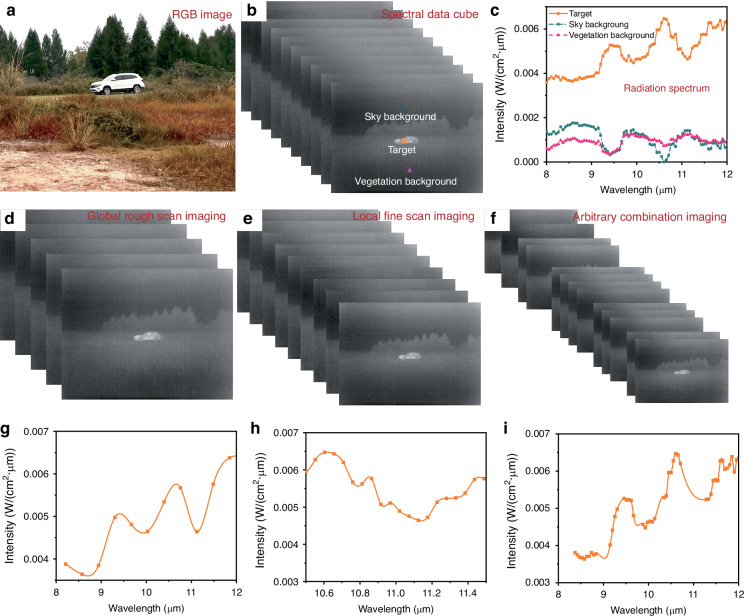
Programmable and adaptive spectral imaging validation of CASI. **a** RGB image of the scenario; **b** Spectral data cube from global fine scanning with corresponding **c** scenario spectral profile; **d**–**f** Data cubes from global coarse scanning, localized fine scanning, and random channel combination imaging; **g**–**i** Spectral signatures derived from global coarse scanning, localized fine scanning, and random channel combination imaging, respectively

For real-world deployment, the CASI was validated through LWIR spectral imaging of vehicle targets. An RGB image of the scenario is presented in Fig. 5a. Initial full spectral data acquisition employed global fine scanning (current step: 2 mA) to capture spectral data cube for subsequent channels selection and target detection algorithm training, with representative channel images shown in Fig. 5b. Processing of these data cubes yielded high-resolution spectral signatures (Fig. 5c), revealing distinct spectral contrasts between vehicles versus sky/vegetation backgrounds. Programmable control of spectral channels via the cooperative matching system enabled acquisition of spectral data cubes (Fig. 5d–f) and corresponding spectral signatures (Fig. 5g–i) across multiple operational modes. Increasing the current step to 20 mA facilitated global coarse scanning (Fig. 5d), which substantially reduced imaging time despite yielding lower-resolution vehicle spectral signatures (Fig. 5g). Targeted refinement within the 10.5–11.5 μm feature band generated localized high-definition spectral image series and precise spectral profiles (Fig. 5e, h), enabling rapid extraction of characteristic spectral information for quantitative target analysis. To validate programmable channel combinations, randomly selected spectral channels within 8–12 μm were configured, producing integrated data cubes (Fig. 5f) and spectral signatures (Fig. 5i). This mode simultaneously delivers rapid global spectral surveys and localized high-fidelity data. Collectively, these results demonstrate CASI’s programmable multi-mode spectral imaging capabilities, featuring customizable spectral resolution, operational wavebands, and data cube configurations. Such flexibility supports diverse application-specific imaging requirements, fulfilling the “multifunctional instrument” paradigm.

#### Efficient target detection via adaptive spectral channel combination imaging

**Fig. 6:**
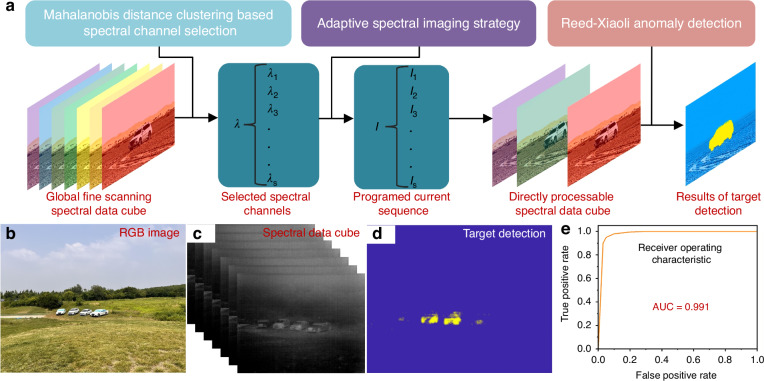
Target detection verification based on adaptive spectral imaging. **a** Schematic of target detection via spectral channel combination imaging; **b** The arranged genuine-decoy vehicle scenario; **c** Data cube acquired through spectral channel combination imaging; **d** Detection results using the RX detector; **e** Performance evaluation of the RX detector

By harnessing the programmable adaptive capabilities inherent of the CASI, coupling with spectral channel selection and target detection algorithms^24^, highly efficient and precise target detection and identification can be achieved, as evidenced by Fig. 6a. Initially, a Mahalanobis distance clustering based spectral channel selection method is applied to the acquired comprehensive hyperspectral spectral data cube. Subsequently, the selected spectral channels are programmed in the synergistic adaptation control system, directly yielding a dimensionally reduced spectral image spectral data cube of the target scenario. Finally, the Reed-Xiaoli (RX) anomaly detection methodology performs target detection directly on this data cube to identify targets. This method effectively reduces data redundancy, streamlines the target detection workflow, and enhances detection accuracy and effectiveness.

Spectral channel selection for the scenario in Fig. 5 was performed using the Mahalanobis distance clustering-based spectral channel selection method, yielding the optimal spectral sequence ***λ***:

$${\boldsymbol{\lambda }}=\{8.184{\rm{\mu }}{\rm{m}},\,8.319{\rm{\mu }}{\rm{m}},\,9.399{\rm{\mu }}{\rm{m}},\,9.804{\rm{\mu }}{\rm{m}},\,10.614{\rm{\mu }}{\rm{m}},\,11.109{\rm{\mu }}{\rm{m}},\,11.604{\rm{\mu }}{\rm{m}}\}$$

The calibrated *λ–I* relationship was used to derive the corresponding drive current sequence ***I***:

$${\boldsymbol{I}}=\{-82{\rm{mA}},-76{\rm{mA}},-28{\rm{mA}},-10{\rm{mA}},26{\rm{mA}},48{\rm{mA}},70{\rm{mA}}\}$$

This sequence was programmed into the synergistic adaptation control system. Validation employed a genuine-decoy vehicle scenario (Fig. 6b), where the conventional RGB imaging failed to discriminate two genuine targets from a distance. Direct imaging with the selected spectral channels produced a dimensionally reduced spectral data cube (Fig. 6c) containing the channel-specific image series. Application of the RX detection algorithm to this data cube enabled direct spectral fusion, successfully identifying genuine targets in the fused output (Fig. 6d, e). The fusion process produced a receiver operating characteristic (ROC) curve with an area under the curve (AUC) of 0.991, demonstrating high detection accuracy.

These experimental results confirm that the CASI exploits the MEMS-FPFC’s programmable filtering strengths to satisfy heterogeneous spectral imaging requirements via adaptive capabilities. Beyond matching the high-fidelity spectral acquisition performance of traditional LWIR spectral imager, CASI enables on-demand spectral channel configuration for target-specific detection scenarios, overcoming the inherent limitations of fixed-channel systems suffer from spectral inflexibility and narrow applicability. Critically, the compact and lightweight design of CASI facilitates deployment on small unmanned platforms. In future work, we will focus on advancing the intelligent software algorithms for the CASI system. By leveraging the complementary strengths of algorithmic intelligence and adaptive hardware, we aim to realize LWIR spectral imaging capable of adaptive detection in response to changing scenes. Furthermore, we will focus on the engineering performance evaluation of the complete CASI system, including its expected operational lifetime and actuation cycle constraints, to ensure its suitability for diverse application scenarios. This integrated approach is expected to significantly broaden the application of LWIR spectral imaging in both civilian and defense-related fields.

## Conclusion

Collectively, we reported the miniaturized LWIR spectral imager based on MEMS-FPFC firstly to the best of our knowledge, achieving programmable and adaptive spectral imaging capabilities. Termed CASI, this system employs a chip-fronted optical design that enables plug-and-play functionality while significantly reducing volume (15 × 7.7 × 8.8 cm³) and weight (1.11 kg), thereby facilitating deployment on lightweight unmanned platforms. Arbitrary spectral channel programming is enabled by a synergistic adaptation control system, which supports multiple spectral imaging modes, including fine scanning, coarse scanning, and arbitrary spectral channel combination. This programmability overcomes the fixed-mode limitations of conventional spectral imagers, adapting the system to diverse application requirements. Following calibration, CASI acquires mode-specific spectral signatures across its operational range. When integrated with channel selection methods and image fusion algorithms, the system achieves efficient and precise target detection. Exploiting the strengths of CASI’s hardware intelligence and computational optics could enable intelligent perception in mineral exploration, gas-liquid detection, and military reconnaissance/early warning.

## Supplementary information

Supplementary Information 1

Supplementary Information 2
