# Supplementary material for: Compact adaptive spectral imager enabled by MEMS Fabry-Perot filtering chip in longwave infrared
Source: Microsyst Nanoeng. 2026 May 26;12:207. doi: 10.1038/s41378-026-01300-6 (PMC13212909; doi:10.1038/s41378-026-01300-6)
Supplement: Supplementary file 1 — Supplementary Information 1 [file 41378_2026_1300_MOESM1_ESM.docx]

**Supplementary Information 1**

**Compact Adaptive Spectral Imager Enabled by MEMS Fabry-Perot Filtering Chip in Longwave Infrared**

*Kui Zhou^1, 2^, Xiaodong Wang^1, 2^, Geng Tong^1, 2^, Xingchen Xiao^1, 2^,*

*Jiancun Zhao**^1,2^, Xiaochang Yu^1,2^ and Yiting Yu^1, 2,^ **

*1 School of Mechanical Engineering, Ningbo Institute of Northwestern Polytechnical University, Key Laboratory of Micro/Nano Systems for Aerospace (Ministry of Education), Key Laboratory of Micro- and Nano-Electro-Mechanical Systems of Shaanxi Province, Northwestern Polytechnical University, 127 Youyi West Road, Xi’an, 710072 China*

*2 Key Laboratory of Scale Manufacturing Technologies for High-Performance MEMS Chips of Zhejiang Province, Key Laboratory of Optical Microsystems and Application Technologies of Ningbo City, 218 Qingyi Road, Ningbo, 315103 China*

**Correspondence: Yiting Yu (E-mail:* [*yyt@nwpu.edu.cn*](mailto:yyt@nwpu.edu.cn)*; Tel: +86-29-88460353-617; Fax: +86-29-88495102)*

**Abstract：**This supplementary information presents theoretical analyses and experimental results on the filtering stability of electromagnetically actuated MEMS-FPFC, including filtering repeatability tests and environmental influence tests.

**1. Filtering repeatability test results**

To ensure stable and precise acquisition of target spectral information across diverse filtering modes, the MEMS-FPFC device must exhibit excellent filtering stability. We therefore conducted tests on its filtering repeatability and linear regression performance. When measuring the central wavelength repeatedly at actuating currents of 0 mA, -60 mA, and 60 mA with distinct current step sizes (2 mA, 10 mA, and 20 mA). Results shown in Fig. S1-1(a), the maximum recorded transmittance (*T*) error was 0.6%, which likely originates from random sources such as instrumental variability or environmental factors (e.g., temperature and vibration). Given that the MEMS‑FPFC achieves a relatively high transmittance of 63.8%-76.2%, this absolute error corresponds to a relative error of only 0.79%-0.94%, a negligible magnitude. Consequently, even without considering other calibration errors, the resulting radiometric calibration uncertainty remains below 1%. Additionally, The MEMS-FPFC demonstrates a linearity of 98.98%. Repeatability tests reveal a maximum wavelength error of 54 nm. Although this value slightly exceeds the single‑measurement result of 51.41 nm, it remains well within the design tolerance of 80 nm.

Moreover, the linear regression test results for the MEMS-FPFC are presented in Fig. S1-1 (b) and (c). Comparative analysis of spectral responses under forward (current swept from -100 mA to 100 mA) and reverse (current swept from 100 mA to -100 mA) actuating modes revealed robust consistency across spectral range, with minor deviations observed solely within the 7-8 μm waveband. Collectively, these results demonstrate the robust filtering stability of the chip under varied operational modes, providing critical assurance for accurate spectral data acquisition in diverse imaging scenarios.


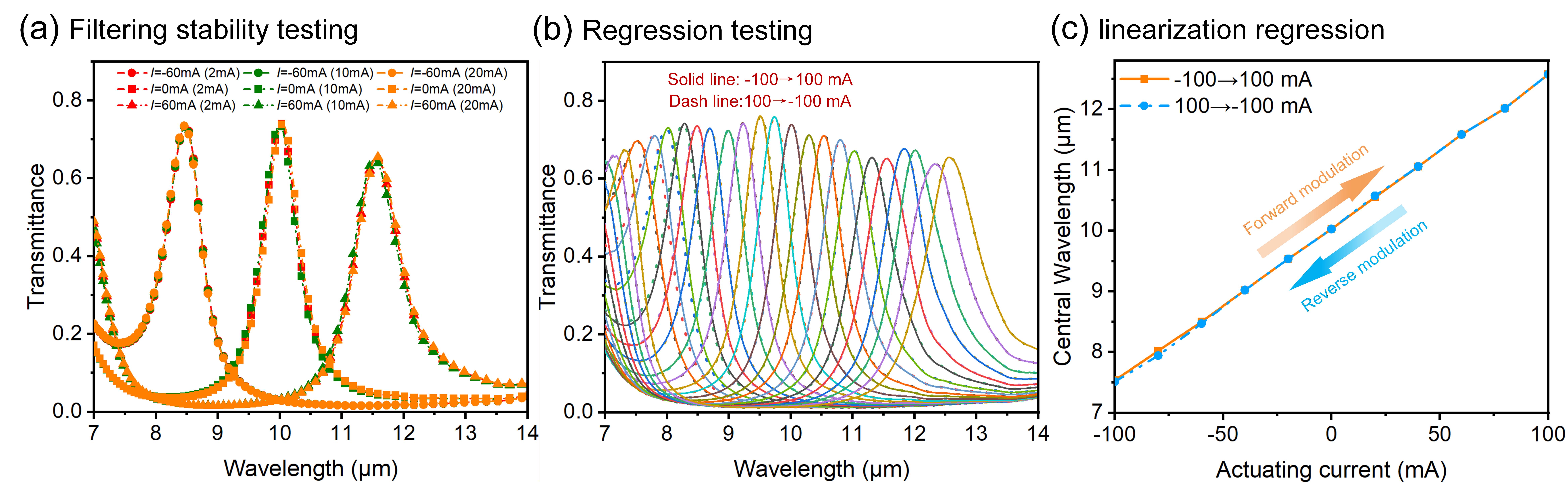


Fig. S1-1: (a) The filtering stability testing and (b)-(c) the regression testing results indicates the MEMS-FPFC has a robust filtering stability.

**2. Environmental influence test results**

The MEMS-FPFC is a filtering chip incorporating a dynamically tunable structure, whose spectral characteristics are inherently susceptible to environmental factors, primarily temperature and vibration. To evaluate these influences, temperature drift and forced-vibration tests were conducted on the device. For the temperature assessment, tests were performed over an operational range of 20-60 °C, with the room-temperature 20 °C as the reference. An electric heating film was attached to the PCB actuating circuit of the MEMS-FPFC to precisely control the temperature, as presented in Fig. S1-2(a). The wavelength shift (*∆λ*) as a function of temperature (*T*) is shown in Fig. S1-2(b). Owing to the use of materials with linear thermal expansion coefficients within this temperature interval, *∆λ* exhibits a linear relationship with *T*, with a maximum observed drift of 37.9 nm. Moreover, across this temperature range, the transmittance varied irregularly between 78.44% and 79.31%, representing a change of less than 1%.

The temperature drift discussed above originates primarily from ambient environmental conditions. Although the MEMS-FPFC employs a wound coil as its electromagnetic actuator, coil self‑heating was addressed at the design stage. A low‑resistance coil (7.8 Ω measured) was selected, and the catuating current is limited to below 100 mA, resulting in a maximum coil dissipation of only 78 mW. This negligible heat output does not affect the performance of the MEMS‑FPFC.


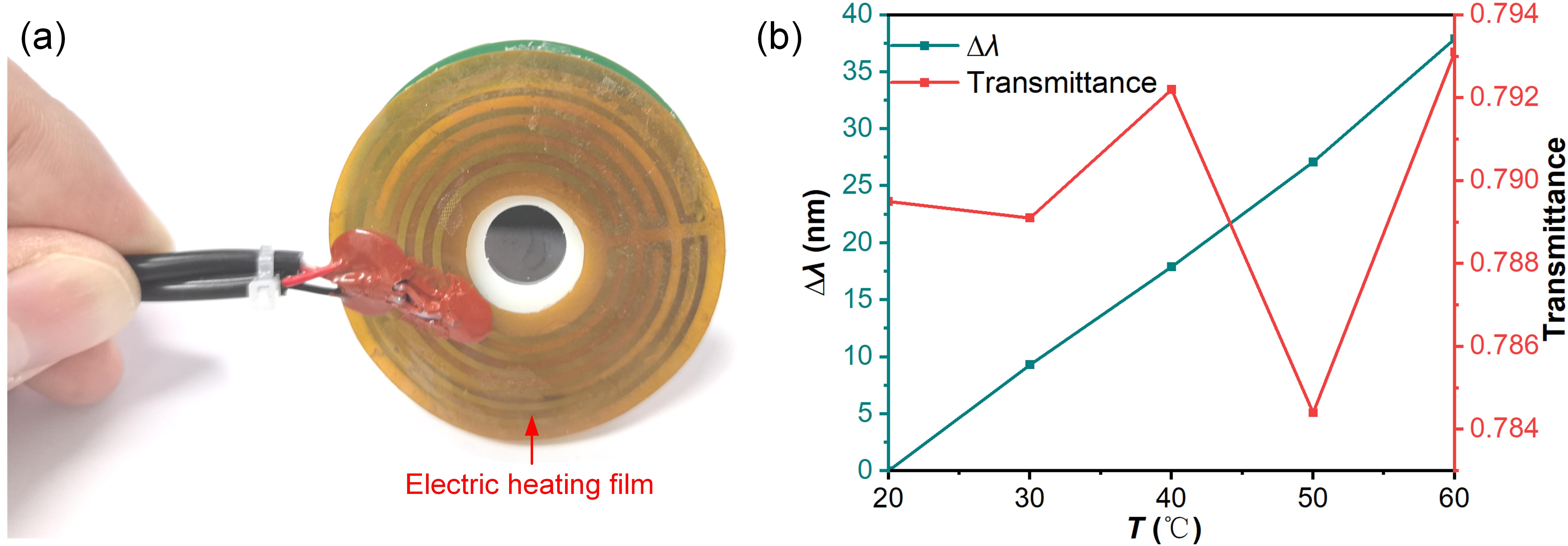


Fig. S1-2: The temperature drift test of MEMS-FPFC: (a) the electric heating film attached onto the MEMS-FPFC, (b) the temperature drift test results under different temperature.

In anticipated applications such as spectral imaging onboard unmanned aerial vehicles (UAVs), platform-induced vibration may drive forced oscillations in the MEMS-FPFC. To quantify this effect, the vibration profile of a commercial UAV (DJI, M600 Pro) was firstly measured using a vibrometer (MSR Electronics GmbH, MSR 165), as shown in Fig. S1-3 (a). The gimbal vibration frequency was found to range from 102.4 Hz to 157.5 Hz. Based on this result, a forced-vibration test was designed using a piezoelectric ceramic plate as the excitation source. The chip was mounted onto the piezoelectric ceramic plate, which was driven by a function generator with a swept-frequency signal from 0 Hz to 600 Hz. The resulting displacement of the chip was monitored with a laser doppler vibrometer (Sunny Optical Technology (Group) Company Limited, LV-S01-M). The first resonant frequency of the MEMS-FPFC was identified at 212.4 Hz. Below this frequency, the chip undergoes translational motion. Above 212.4 Hz, the vibration mode shifts to a torsional movement, leading to MEMS-FPFC device failure. Within the working frequency band of 0-180 Hz, the vibration amplitude remained below 24.5 nm, corresponding to a filtering error of 49.0 nm.


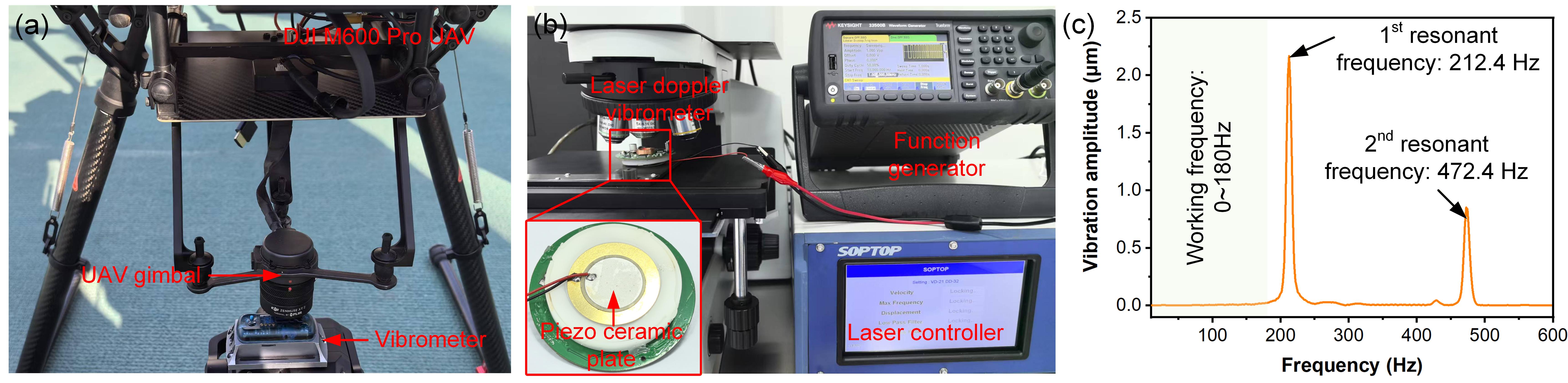


Fig. S1-3: The vibration impact test of the MEMS-FPFC: (a) vibration characterization of the UAV Platform, (b) forced vibration testing via laser doppler vibrometer, and (c) the test result.

Additionally, the incident angle of incoming light influences the filtering performance. According to the Fabry-Perot (FP) principle governing the MEMS-FPFC:

$\lambda=\frac{2nd\cos\theta}{m}$ (S1-1)

where *n*, *d*, *m* are the refractive index of the FP cavity medium, the FP cavity length, the interference order, and *θ* is the incident angle. Here, both n and m are equal to 1, the Eq. (S1-1) can be simplified to:

$\lambda=2d\cos\theta$ (S1-2)

Deviations in *θ* thus induce a blue shift in the transmitted wavelength, an inherent feature of the MEMS-FPFC. The filtering response was measured under varying *θ*, as summarized in Fig. S1-4. The tests were performed at three center wavelengths: approximately 8μm, 10μm, and 12 μm. Over a 14° field of view (FOV) of the CASI, the wavelength shift (*∆λ*) ranged from 236.31 nm to 358.81 nm, which is substantially greater than the linearity error of the MEMS-FPFC itself.

In designing the CASI architecture, we evaluated several optical layouts to balance performance and practicality. Two primary alternatives were examined: placing the MEMS‑FPFC within an intermediate focal plane, or locating it between the imaging lens and the detector. The first configuration would necessitate a long optical path with a large aperture to accommodate the PCB‑packaged MEMS‑FPFC under near‑normal incidence. Even with a clear aperture of 11 mm, the device would obstruct a substantial portion of the beam, significantly reducing optical throughput. The second arrangement, while avoiding central obscuration, would allow focused light to reach the detector with a wide angular distribution. This would introduce strong angular‑dependent spectral shifts and complicate optical alignment and system integration. After thorough comparison, we adopted a chip‑in‑front architecture. This layout makes full use of the MEMS‑FPFC’s large clear aperture, yielding a compact system with a short optical path and high throughput. The trade‑off is a known spectral sensitivity to off‑axis angles, which we mitigate in practice by positioning targets near the centre of the field of view. The design rationale is further detailed in the revised manuscript.


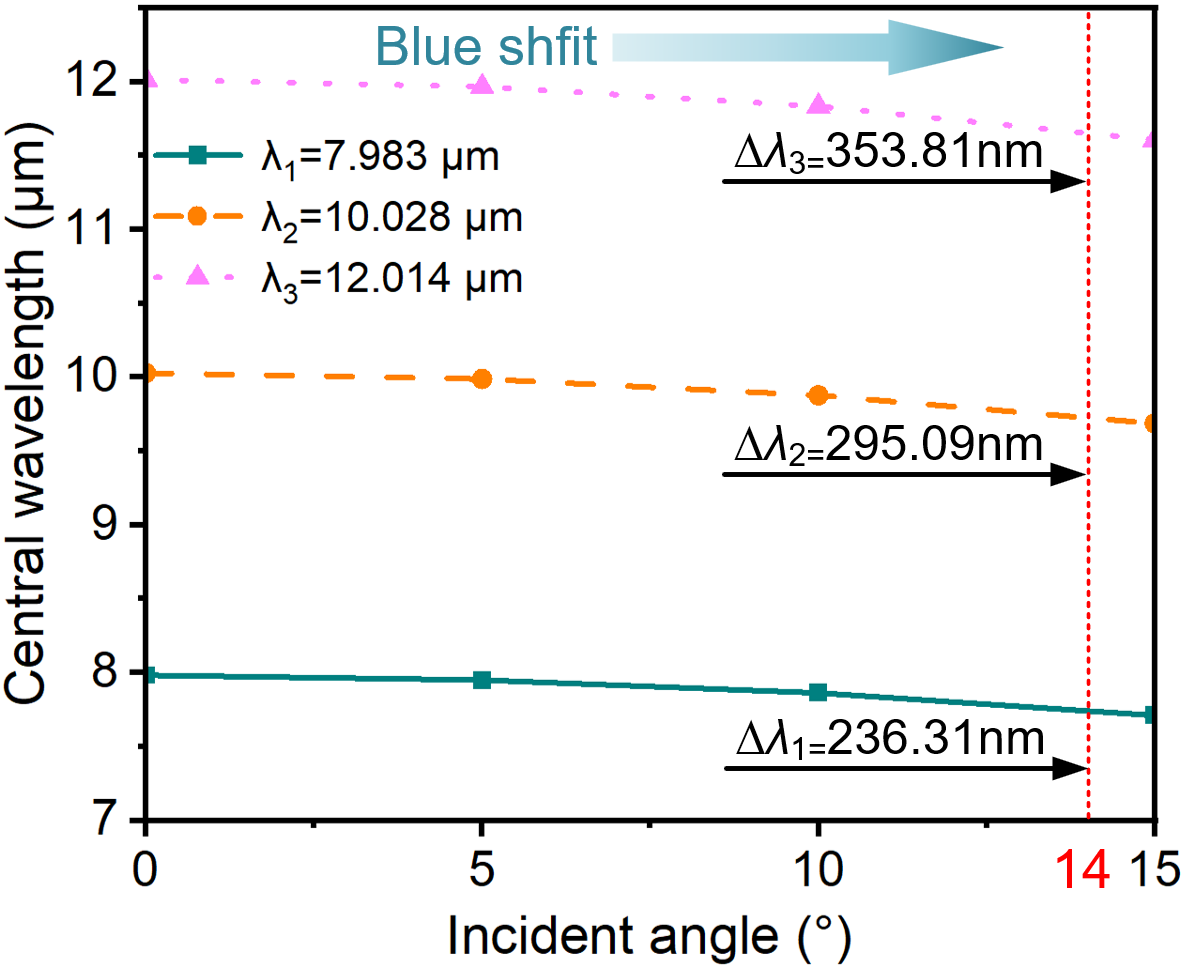


Fig. S1-4: The characterization of incident angle vs wavelength shift of the MEMS-FPFC
