# Supplementary material for: Compact adaptive spectral imager enabled by MEMS Fabry-Perot filtering chip in longwave infrared
Source: Microsyst Nanoeng. 2026 May 26;12:207. doi: 10.1038/s41378-026-01300-6 (PMC13212909; doi:10.1038/s41378-026-01300-6)
Supplement: Supplementary file 2 — Supplementary Information 2 [file 41378_2026_1300_MOESM2_ESM.docx]

**Supplementary Information 2**

**Compact Adaptive Spectral Imager Enabled by MEMS Fabry-Perot Filtering Chip in Longwave Infrared**

*Kui Zhou^1, 2^, Xiaodong Wang^1, 2^, Geng Tong^1, 2^, Xingchen Xiao^1, 2^,*

*Jiancun Zhao**^1,2^, Xiaochang Yu^1,2^ and Yiting Yu^1, 2,^ **

*1 School of Mechanical Engineering, Ningbo Institute of Northwestern Polytechnical University, Key Laboratory of Micro/Nano Systems for Aerospace (Ministry of Education), Key Laboratory of Micro- and Nano-Electro-Mechanical Systems of Shaanxi Province, Northwestern Polytechnical University, 127 Youyi West Road, Xi’an, 710072 China*

*2 Key Laboratory of Scale Manufacturing Technologies for High-Performance MEMS Chips of Zhejiang Province, Key Laboratory of Optical Microsystems and Application Technologies of Ningbo City, 218 Qingyi Road, Ningbo, 315103 China*

**Correspondence: Yiting Yu (E-mail:* [*yyt@nwpu.edu.cn*](mailto:yyt@nwpu.edu.cn)*; Tel: +86-29-88460353-617; Fax: +86-29-88495102)*

**Abstract：**In this supplement information, we detail the spectral and radiometric calibration methods for CASI, along with the data processing workflow and results, in support of the calibration findings presented in the main text.

**1. Spectral calibration of CASI**

Although the filtering spectral of the MEMS‑FPFC filter chip was characterized in advance, establishing a well‑defined linear relationship of *λ‑I*, deviations inevitably arise during CASI system‑level operation. Factors such as non‑uniform transmission across the optical path and variations in the incident angle introduce discrepancies between the spectral response measured at the chip level and that ultimately incident on the detector. Consequently, spectral calibration of the complete imaging system is essential. For this purpose, we adopted the “characteristic spectral‑band method” to calibrate the CASI. This approach employs a light source with known spectral features as monochromatic input. The spectral imaging system scans across the wavelength range, and the resulting digital number (DN) values are analyzed to identify the wavelength corresponding to the peak response-indicating alignment with the input monochromatic line. This method is particularly well suited to spectral imaging systems that support continuous scanning across multiple spectral channels.

Given the large number of spectral channels in the CASI system, calibrating each channel individually would substantially increase the workload. However, the filtering performance of the MEMS‑FPFC, particularly the linear relationship between the central wavelength (*λ*) and the actuating current (*I*), has already been characterized in prior device‑level tests. To reduce the calibration effort, we therefore selected a subset of representative spectral channels for system‑level calibration and compared the resulting deviations between the calibrated spectra and the chip‑level test data. The spectral calibration setup is illustrated in Supplementary Fig. S2‑1(a). A monochromator (Zolix, Omni‑λ500i) was used as the primary light source to generate monochromatic output. An integrating sphere was attached to the exit port of the monochromator to homogenize and collimate the beam. The CASI under test was aligned with the output port of the integrating sphere. A series of spectral channels across the system’s operating spectral range were selected as input values for the monochromator, denoted as:

$\lambda’=\{\lambda’_{1}\text{，}\lambda’_{2}\text{，}\lambda’_{3}\ldots\}$ (S2-1)

The CASI system performed global fine scanning spectral imaging across the full range for each output spectral channels *λ*’, generating corresponding spectral image data cubes. From each data cube, the digital number (DN) value curve was extracted and fitted using a Gaussian function. The actuating current corresponding to the peak of the fitted curve was then determined as:

$I=\{I_{1}\text{，}I_{2}\text{，}I_{3}\ldots\}$ (S2-2)

As shown in Fig. S2‑1(b), the corresponding central wavelength under test was then calculated by substituting this current value into the *λ‑I* linear relationship obtained from prior spectral characterization, denoted as:

$\lambda=\{\lambda_{1}\text{,}\lambda_{2}\text{,}\lambda_{3}\ldots\}$ (S2-3)

As shown in Fig. S2-1(c). The deviation between the output spectral channels *λ*’ from the monochromator and the central wavelength *λ* obtained from MEMS-FPFC testing, for a given actuating current, is as follows:

$\Delta\lambda=\left(\lambda-\lambda’\right)/\lambda=\{\Delta\lambda_{1}\text{，}\Delta\lambda_{2}\text{，}\Delta\lambda_{3}\ldots\Delta\lambda_{n}\}$ (S2-4)

The nonlinearity error *L’* resulting from this deviation was then calculated as follows:

$L'=1-\Delta\lambda/FSR$ (S2-5)

A decision rule based on the comparison between the calculated nonlinearity error *L’* and the chip linearity *L* governs the calibration outcome: When *L’* ≥ *L*, the calibration error is within the chip’s tolerance; the chip-characterized *λ* is accepted as the calibration reference, and the spectral resolution is defined by the minimum adjacent-channel spacing. When *L’* falls significantly below *L*, indicating integration-induced spectral distortion, the monochromator output *λ’* becomes the reference. A stepwise recalibration of the system’s central wavelengths is then performed, with spectral resolution recomputed from the recalibrated channel intervals. All subsequent radiometric and spectral imaging procedures rely on these calibrated channel definitions.


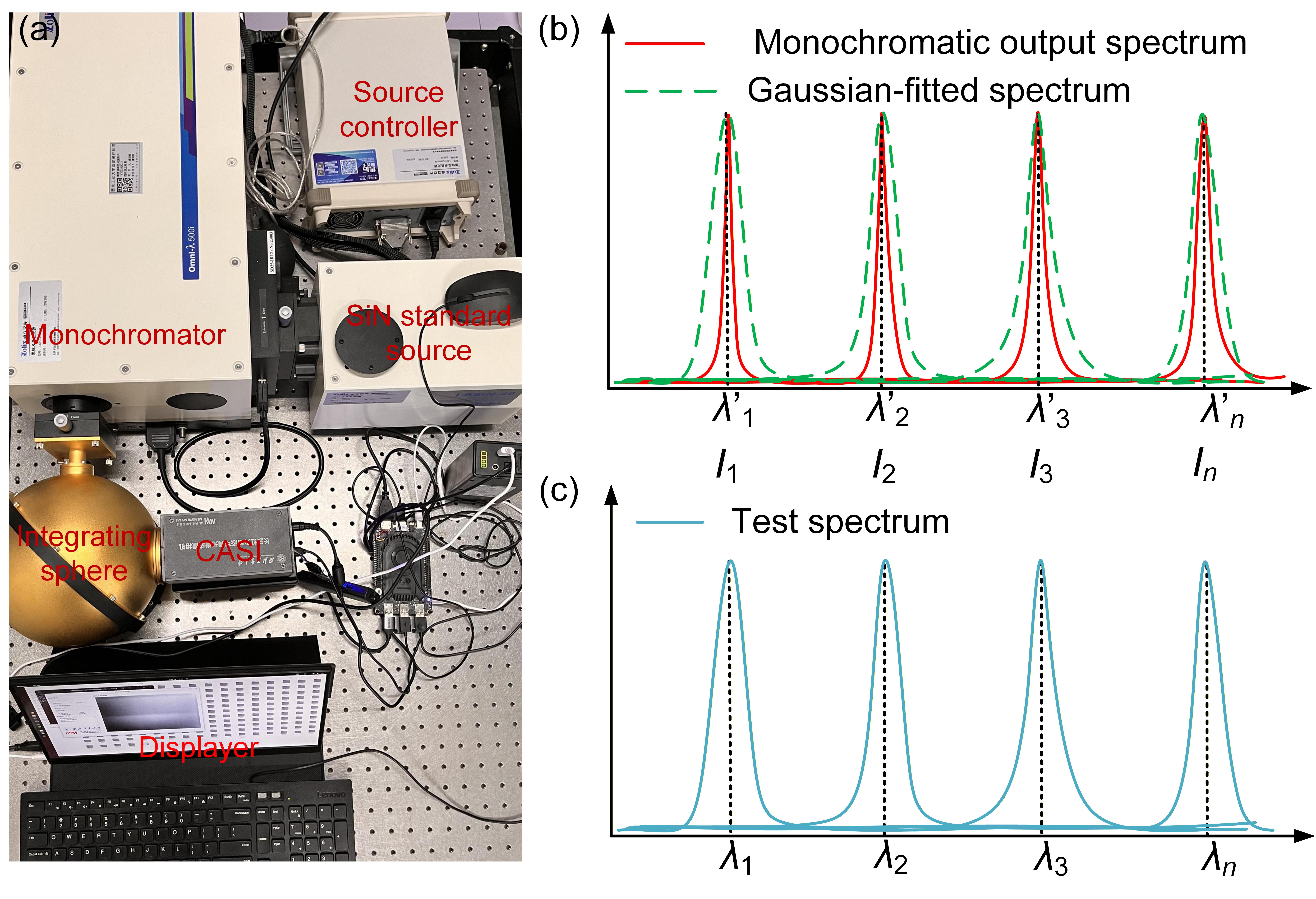


Fig. S2-1. Schematic diagram of the CASI spectral calibration method: (a) Configuration of the spectral calibration system; (b) The monochromator outputs *λ’*. After imaging by the CASI, Gaussian fitting is applied to the data to determine the actuating current value corresponding to the peak wavelength; (c) The filtering *λ* of the MEMS-FPFC is calculated by substituting this current value into the λ-I formula.

Calibration channels were selected at 0.5 μm intervals across the 8-12 μm band. Applying the above calibration procedure yielded the results summarized in Table S2‑1. The maximum wavelength deviation observed was 44 nm, corresponding to a nonlinearity error *L*’ of 99.13% for the MEMS‑FPFC under this worst‑case condition. This indicates that the calibration error remains within the intrinsic linearity tolerance of the MEMS-FPFC. Consequently, the *λ* determined from the MEMS-FPFC chip‑level characterization were adopted as the spectral calibration reference. According to the *λ‑I* linear relationship:

$$\lambda=0.0225*I+10.029$$

CASI achieves a minimum spectral sampling interval of 22.5 nm. Subsequent radiometric calibration of the system was performed based on the spectrally calibrated channel positions established above.

Table S2-1. Comparison of spectral calibration data across different channels

| *λ*’（μm） | 8 | 8.5 | 9 | 9.5 | 10 | 10.5 | 11 | 11.5 | 12 |
| --- | --- | --- | --- | --- | --- | --- | --- | --- | --- |
| *I*(mA) | -92 | -66 | -44 | -25 | -1 | 19 | 44 | 67 | 86 |
| *λ*（μm） | 7.959 | 8.544 | 9.039 | 9.467 | 10.007 | 10.457 | 11.019 | 11.537 | 11.964 |
| *Δλ*（nm） | 41 | 44 | 39 | 33 | 7 | 43 | 19 | 37 | 36 |

**2. Radiometric calibration of CASI**

Radiometric calibration of the CASI system was performed using the “two-point method”, as illustrated in Fig. S2‑2. A blackbody source provided a standard spectral radiance, which was collimated into a parallel beam via an integrating sphere. The CASI acquired global fine scanning spectral images of this beam, yielding a spectral data cube *DN*(*λ*). The corresponding theoretical blackbody intensity spectrum *Q*(*λ*) was calculated using Planck’s law. By setting two standard radiance levels- a low value *Q*_1_(*λ*) and a high value *Q*_2_(*λ*), and acquiring spectral images at each level, the following relationship was obtained:

 (S2-6)

In this case, the parameters *Q*_1_(*λ*), *Q*_2_(*λ*), *DN*_1_(*λ*) and *DN*_2_(*λ*) are all known. By solving the system of two linear equations, the gain coefficient *G*(*λ*) and offset coefficient *O*(*λ*) for each spectral channel can be determined.


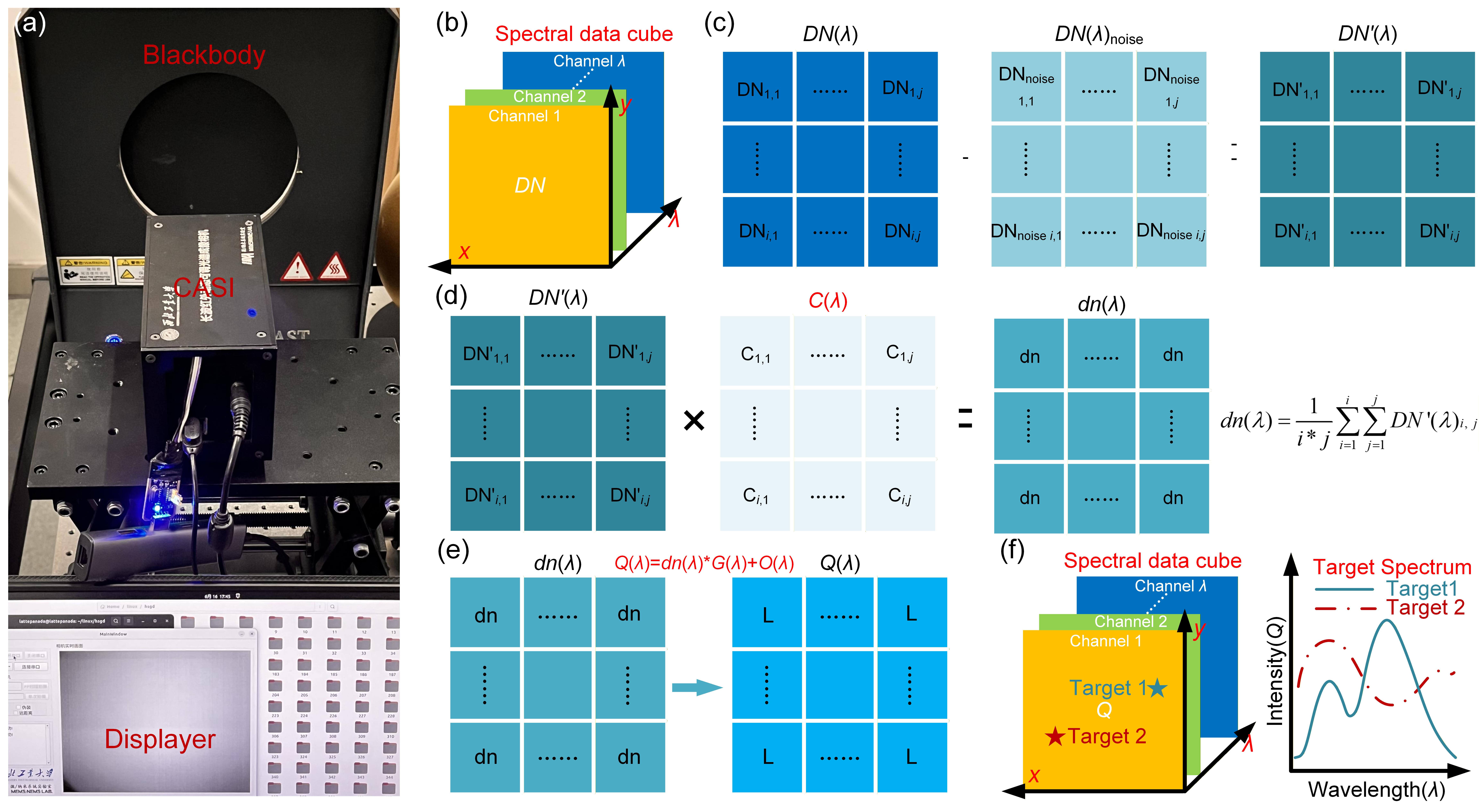


Fig. S2-2. Radiometric calibration and spectral data processing workflow for the CASI: (a) Configuration of the radiometric calibration system; (b) Spectral data cube; (c) Dark noise correction; (d) Non-uniformity correction; (e) Absolute radiometric calibration; (f) Spectral information extraction.

The radiometric calibration of a spectral imaging system typically comprises three sequential steps: dark-noise (thermal noise) correction, flat-field correction (non-uniformity correction), and absolute radiometric calibration. Each of these procedures essentially involves processing the spectral image data. The workflow and corresponding data processing stages are illustrated in Fig. S2‑2(b)-(f). Firstly, the “two-point calibration method” is employed to acquire raw spectral data cubes at high and low radiance levels (Fig. S2‑2(b)). These data cubes contain spectral images with *i* × *j* pixels for each spectral channel. Taking a representative spectral channel *λ* as an example, the response of each pixel in its spectral image can be expressed as a matrix *DN*(*λ*), which consists of two superimposed components: the true pixel response *DN*’(*λ*) and the dark-noise response *DN*’(*λ*)noise. The dark-noise component *DN*’(*λ*)_noise_ typically arises from multiple sources, including the dark current of the detector pixels, dark current in the detector driving circuitry, and thermal noise from various parts of the imaging system. Its magnitude can be characterized by performing spectral imaging measurements in a dark room with no optical input, allowing it to be removed as an additive factor. Therefore, the first step in radiometric calibration is to eliminate the influence of dark current through dark-noise (thermal noise) correction (Fig. S2‑2(c)). In terms of image data processing, this is achieved by image subtraction to obtain the pure pixel response *DN*’(*λ*):

$DN'(\lambda)=DN(\lambda)-DN(\lambda)_{noise}$ (S2-7)

After *DN*’(*λ*)_noise_ removal, the raw responses of individual pixels to the same radiance level still exhibit variations due to factors such as optical system non-uniformity, detector response inhomogeneity, and differences in operating conditions. These residual discrepancies necessitate non-uniformity correction of the system. As illustrated in Fig. S2‑2(d), the objective of this correction is to normalize the pixel responses across the image to a uniform value, specifically, the mean response *dn*(*λ*) of all pixels within the image. This reference value is defined as:

 (S2-8)

Therefore, under the constraint that the mean response remains unchanged, the non-uniformity correction coefficient matrix *C*(*λ*) for each pixel can be calculated as follows:

$DN'(\lambda)*C(\lambda)=dn(\lambda)$ (S2-9)

After correction, the pixel responses become uniform across the image. The non-uniformity correction coefficient matrices for each spectral band are recorded. Finally, based on the corrected pixel response value *dn*(*λ*), the *G*(*λ*) and offset coefficient *O*(*λ*) for each channel are calculated as follows:

 (S2-10)

Through the calibration procedure outlined above, performed sequentially for each spectral band, the corresponding spectral response functions are derived as:

$\begin{matrix} Q(1)=dn(1)*G(1)+O(1) \\ Q(2)=dn(2)*G(2)+O(2) \\ . \\ . \\ . \\ Q(\lambda)=dn(\lambda)*G(\lambda)+O(\lambda) \end{matrix}$ (S2-11)

Substituting into Equation (S2-7) yields:

 (S2-12)

Following this calibration procedure, the spectral imaging system acquires the capability to extract differential spectral information of targets from the original three-dimensional spectral data cube, as illustrated in Fig. S2‑2(e). In subsequent practical spectral imaging applications, the processing workflow for spectral data cubes acquired from real scenes follows the same sequence outlined in Supplementary Fig. S2‑2. This involves first removing dark noise from the images, followed by non-uniformity correction, and finally computing the spectral information using the spectral response function given in Equation (S2‑12).

Radiometric calibration was performed using blackbody sources at 35 °C and 100 °C as the low‑ and high‑temperature reference points, respectively. To validate the accuracy of the calibrated spectral imaging system in extracting spectral information, we subsequently imaged blackbody sources at 55 °C and 112 °C and retrieved their radiance spectra. The results are presented in Fig. S2‑3. Owing to the emissivity of the blackbody sources (0.95-0.97), which is slightly below the ideal value of unity, and without accounting for the system's self‑emitted radiation, the extracted spectral radiance values are marginally lower than the theoretical spectra calculated from Planck's law. The root mean square errors (RMSE) at 55 °C and 112 °C were 0.00029 W/(cm²·μm) and 0.000262 W/(cm²·μm), respectively. Overall, the experimentally extracted spectra show good agreement with theoretical predictions, thereby validating the effectiveness of the proposed calibration method.


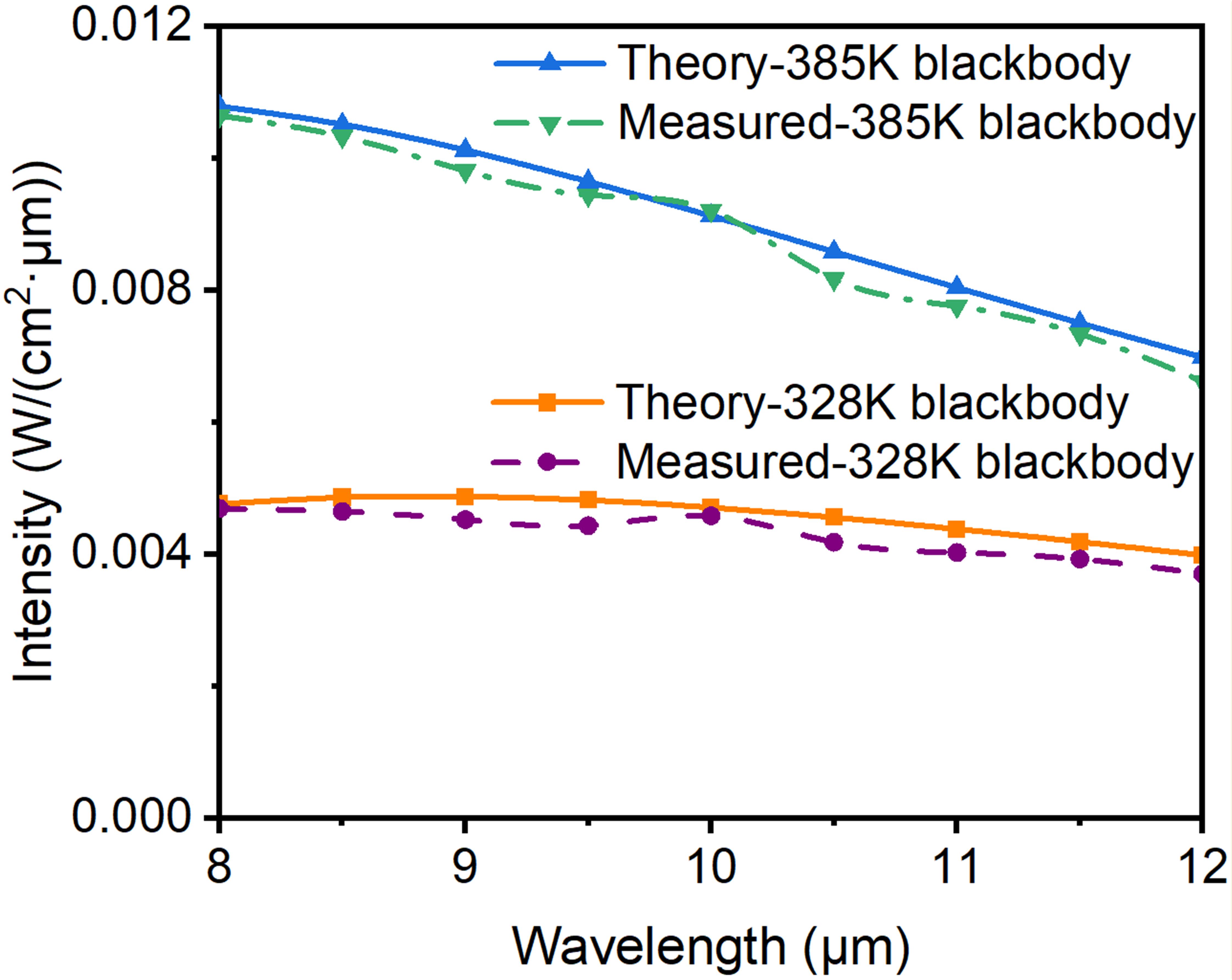


Fig. S2-3. Calibration of the CASI system using a blackbody for imaging validation

To further evaluate the imaging performance of the CASI system, its noise‑equivalent temperature difference (NETD) was characterized using the thermal imaging assessment system, as illustrated in Fig. S2-4. The NETD value was derived from measurements of the signal transfer function (SiTF) and the temporal noise *σ*_TVH_, according to the following expression:

$\text{NETD}=\frac{\sigma_{\text{TVH}}}{\text{SiTF}}$ (S2-13)

Taking the spectral channel centred at the initial wavelength *λ*_0_ as an example, the measurement results are presented in Fig. S2-4(c) and (b). The signal transfer function (SiTF) was determined to be 3.880 Cnts/°C, and *σ*_TVH_ was 4.391 Cnts, yielding an NETD of 1.132 °C. By selectively measuring spectral channels across the 8-12 μm band, the NETD values were found to range from 1.066 to 1.542 °C. This relatively high NETD is primarily attributable to factors inherent to LWIR spectral imaging, including spatial resolution degradation and reduced optical throughput. Future work will focus on optimizing the optical design of the CASI system to further improve its imaging performance.


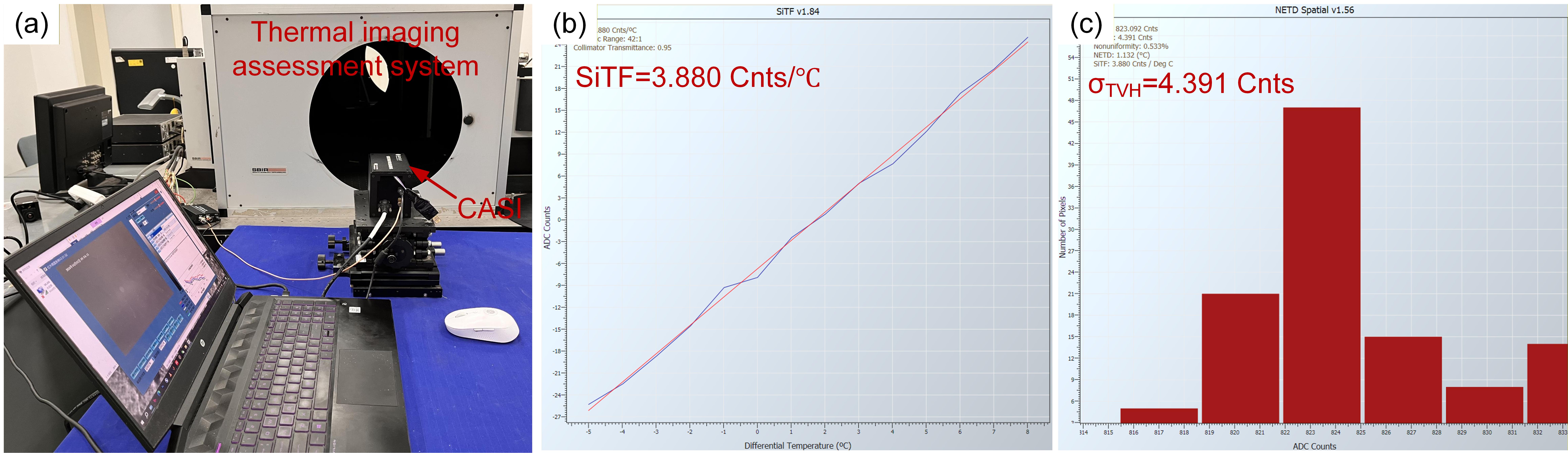


Fig. S2-4. Measurement process of the CASI system’s NETD: (a) NETD measurement setup; (b) and (c) Measurement results of SiTF and *σ*_TVH_.
